# Poisoned by Food

**Published:** 1914-04

**Authors:** 


### Poisoned by Food.

A quarter of a million dead babies, 1120 adult deaths, and 26,582 cases of sickness from eating poisoned foods are enumerated by Rutledge Rutherford in the most remarkable denunciation of food adulteration that has ever appeared.

The list he has compiled looks more like the death roll of a battlefield than a record of food poisonings. In fact, the totals of the casualties among adults amount to more than three times the total American losses in all the battles of the eight years of the revolutionary war, including dead, wounded and captured, for these were only 8000, while the dead and injured from food poisons, enumerated by Mr. Rutherford, amount to 27,702.

A few of the poisonings recorded—not more than 75 or 100—occurred as long ago as five years; all the rest are of recent date, or since the government saw fit to overrule Dr. H. W. Wiley in his action prohibiting the use of chemical preservatives, the writer claims in his article in the current issue of *The National Food Magazine*.

SACRIFICED TO HIGHER PROFITS.

"Think of it, 250,000 babies killed in one year by the food poisoners," says Mr. Rutherford. "A quarter of a million little lives blotted out in the mad race to realize the greatest amount of profit with the least amount of expense. For the only office of chemicals is to cheapen the cost of production."

The total number of officers and men killed in the war of the rebellion was about 190,000. So according to Mr. Rutherford's estimates, 60,000 more little babies were killed by poisoned foods in one year than the number of men killed in all the battles of the Civil War in four years. And if the number of grown people killed by the poisoned diet is included the total from Mr. Rutherford's estimate would reach the appalling figure of 550,000, for he says that one-third of the deaths of adults may be attributed to the same cause.

MANY ARTICLES "TREATED."

Candies colored with coal tar dyes and sweetened with saccharin; soda water with foam produced from soap bark, soothing syrups made deadly with morphine and cocaine; bakery products made of rotten eggs deodorized with chemicals; germ-laden milk with its smell and ill taste concealed with chemicals; pickles containing acetic acid; and mothers' milk poisoned with the chemicals the mother consumes in her foods are among the long list of dangers besetting the child's life, mentioned by the author. And these are the main causes he assigns for the death of the 250,000 babies which, according to his estimate, resulted last year from the eating of adulterated foods.

The author detailed at length the danger to the child in the chemicalized foods the mother eats, warning her against beer, which is doped with salicylic acid, and other alcoholic beverages, many of which are made from wood alcohol distilled from sawdust. He shows that headache powders taken by the mothers often proves disastrous to the child and continues:

"SOOTHING SYRUP DEADLY."

"If the baby becomes ill from the effects of the drugs and chemical poisons its mother consumes, and the mother seeks relief in the chemical concoctions sold in drug stores, then its little life is in peril indeed. Dr. Wiley and his aides have clearly shown that many of the infant remedies sold are deadly poisons. Their supposed soothing effect is due entirely to opiates, even the heinous cocaine, the vilest drug on earth, constituting the basic ingredient of many of them. In a soothing syrup labeled 'a safe and indispensable syrup to make babies quiet and restful,' there was found enough morphine sulphites to kill if given as prescribed on the bottle.

"Thus the child is born into a world of chemicals. But during the nursing period it at least has the protection of its mother's judgment. It is after that and during the time that the child's baby eyes are charmed by the alarming bright colors of candies, foaming soda fountain drinks, frosted bakery cakes and other sweets its nature craves that it is in the greatest danger. Then it is that the chemical tepesters find the little one most susceptible to their wiles and they reap a harvest through its gullibility and immature judgment."—*Bulletin Texas State Board of Health.*

